# Ventricular Tachycardia Following Kratom Ingestion Requiring Extracorporeal Membrane Oxygenation in a Young Woman: Case Report

**DOI:** 10.5811/cpcem.51542

**Published:** 2026-04-21

**Authors:** Megan McLin-Evans, Jenner Tiscareno, Laura Lee Beneke

**Affiliations:** University of Mississippi Medical Center, Department of Emergency Medicine, Jackson, Mississippi

**Keywords:** kratom, ventricular tachycardia, ECMO, cardiac arrest, toxicology, case report

## Abstract

**Introduction:**

Kratom (*Mitragyna speciosa*) is an unregulated herbal supplement increasingly associated with severe toxicity. Concentrated liquid formulations pose risks, with emerging reports of seizures, hepatotoxicity, and arrhythmias.^9^

**Case Report:**

A previously healthy 24-year-old woman ingested a highly concentrated kratom extract and developed seizure-like activity followed by pulseless monomorphic ventricular tachycardia. She underwent approximately 45 minutes of resuscitation, including multiple defibrillations, dual-sequential shocks, amiodarone, lidocaine, magnesium, calcium, sodium bicarbonate, potassium repletion, epinephrine, and esmolol. Persistent instability prompted consultation with cardiology and cardiothoracic surgery, and she was cannulated for venoarterial extracorporeal membrane oxygenation (ECMO) in the emergency department. Lab studies showed profound hypokalemia, acidosis, and elevated lactate. Urine toxicology confirmed mitragynine. She stabilized on ECMO, was decannulated on hospital day two, extubated on day three, and discharged home neurologically intact on day seven.

**Conclusion:**

Concentrated kratom extracts can precipitate life-threatening ventricular arrhythmias in previously healthy individuals. Emergency physicians should consider kratom in unexplained cardiac arrests and recognize the role of advanced support, including ECMO, in refractory toxicologic arrests.

## INTRODUCTION

Kratom, derived from the Southeast Asian tree *Mitragyna speciosa*, has gained increasing popularity in the United States as an over-the-counter supplement marketed for pain relief, mood elevation, and opioid withdrawal.^1^ Its psychoactive alkaloids, primarily mitragynine and 7-hydroxymitragynine, exert both stimulant and opioid-like effects depending on the dose taken.^1^ Despite its widespread availability and natural origin, kratom is not regulated by the U.S. Food and Drug Administration, and commercial preparations often vary significantly in potency and purity.^2^ Reports of toxicity have increased in recent years, including cases involving seizures, hepatotoxicity, QT interval prolongation, and cardiac arrest.^3^,^4^

Concentrated liquid kratom formulations present a particularly high-risk profile due to their potent alkaloid content and ease of ingestion.^5^ Although kratom has not historically been considered arrhythmogenic, growing evidence points to potential pro-arrhythmic properties, particularly in young patients without preexisting cardiac disease.^6^,^7^ Here we describe a case of refractory monomorphic ventricular tachycardia and cardiac arrest following ingestion of a concentrated kratom extract, successfully managed with emergency department (ED)-initiated venoarterial extracorporeal membrane oxygenation (VA-ECMO).

## CASE REPORT

A previously healthy 24-year-old woman was found unresponsive by her boyfriend after reportedly consuming nearly an entire 15-mL bottle of MIT45 Super K Extra Strong (MIT45, Draper, UT), a highly concentrated kratom extract. She reported that she had not slept in several days and developed generalized shaking movements with urinary incontinence before becoming unresponsive. Emergency medical services were called and administered naloxone and midazolam en route to the ED. She was intubated in the field due to ongoing seizure-like activity and altered mental status. Shortly after intubation in the prehospital setting, she became pulseless with a wide-complex rhythm. On arrival to the ED, the patient remained pulseless with a wide-complex monomorphic rhythm consistent with ventricular tachycardia. High-quality cardiopulmonary resuscitation (CPR) was continued. She underwent multiple rounds of standard defibrillation with brief episodes of return of spontaneous circulation on approximately four occasions, each time degenerating back into pulseless ventricular tachycardia within seconds. At no point did she achieve sustained sinus rhythm.

The patient received multiple pharmacologic interventions, including a 300-mg amiodarone bolus with continuous infusion, a lidocaine infusion at 2 mg/minute, 4 grams of magnesium sulfate, 4 grams of calcium chloride followed by 2 grams of calcium gluconate, 175 milliequivalents (mEq) of sodium bicarbonate, aggressive potassium repletion, and multiple 1-mg epinephrine boluses. Due to ongoing electrical instability and concern for electrical storm, an esmolol bolus was given. Despite these measures and continued CPR, she remained in refractory ventricular tachycardia.

Given the lack of sustained return of spontaneous circulation after multiple standard shocks, three dual-sequential defibrillation attempts were performed. None resulted in conversion to a stable perfusing rhythm. After the third unsuccessful dual-sequential shock, cardiology and cardiothoracic surgery were emergently consulted, and the decision was made to proceed with VA-ECMO cannulation in the ED. Laboratory evaluation revealed severe hypokalemia with a potassium level of 1.9 mEq per liter (reference range: 3.5–5.1 mEq/L), sodium 150 mEq/L (135–145 mEq/L), arterial pH 7.18 (7.35–7.45), lactate 12.2 millimoles (mmol) per liter (0.5–2.0 mmol/L), glucose 240 mg per deciliter (dL) (70–110 mg/dL), aspartate aminotransferase 210 units/L (10–40 U/L), alanine aminotransferase 180 U/L (7–56 U/L), and creatine kinase 912 U/L (30–200 U/L). A urine drug screen was positive only for benzodiazepines, consistent with prehospital midazolam administration. Urine toxicology by liquid chromatography-mass spectrometry confirmed mitragynine and its metabolite, along with caffeine, cotinine, fentanyl, lidocaine, midazolam, phenylephrine, and trazodone. Analysis of two MIT45 Super K Extra Strong bottles from the patient’s possession demonstrated mitragynine concentrations of 8.6 mg/mL and 48.3 mg/mL.

*CPC-EM Capsule*

What do we already know about this clinical entity?

*Kratom is an unregulated supplement associated with seizures and toxicity, but life-threatening ventricular arrhythmias are rarely reported*.

What makes this presentation of disease reportable?

*This case describes refractory ventricular tachycardia after kratom ingestion requiring ED-initiated venoarterial extracorporeal membrane oxygenation (ECMO)*.

What is the major learning point?

*High-potency kratom can cause malignant arrhythmias, and early consideration of ECMO may be lifesaving in refractory toxicologic arrest*.

How might this improve emergency medicine practice?

*Awareness of kratom cardiotoxicity could lead to early multidisciplinary activation and ECMO consideration in select arrest patients*.

**Image 1:**
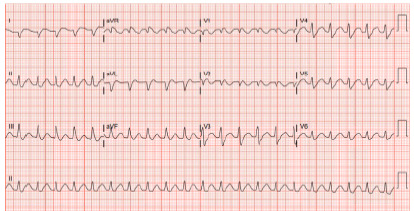
nitial electrocardiogram on arrival of a young woman who ingested kratom, showing wide complex tachycardia with right bundle branch block morphology and a QT interval of 602 milliseconds.

**Image 2:**
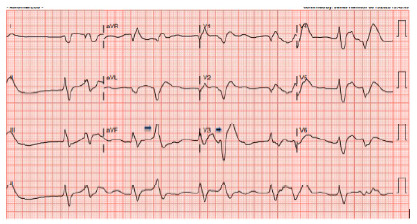
Electrocardiogram demonstrating a ventricular escape rhythm with bigeminal premature ventricular complexes (arrows) and a markedly prolonged QRS interval of 262 milliseconds, consistent with progressive metabolic derangement.

Computed tomography of the head showed no acute intracranial process. Computed tomography of the chest, abdomen, and pelvis demonstrated an acute nondisplaced sternal fracture and fat stranding about the pancreas and duodenum concerning for acute interstitial edematous pancreatitis. Electrocardiography on arrival demonstrated wide complex tachycardia consistent with ventricular arrhythmia in the setting of electrolyte derangement (Images 1 and 2).

The patient was emergently cannulated for ECMO via the left femoral artery and vein at bedside in the ED, with placement of a distal reperfusion cannula after loss of Dopplerable signals in the left foot. Following ECMO initiation, she achieved hemodynamic stability and vasopressor requirements gradually decreased. She was transferred to the cardiovascular intensive care unit (ICU) on multiple vasopressors along with amiodarone and lidocaine infusions. Given concern for vasoplegia, she received methylene blue. N-acetylcysteine and broad-spectrum antibiotics were started for possible toxin-induced organ injury and post-cannulation prophylaxis. Over the first 24 hours in the ICU, she exhibited intermittent generalized myoclonus and perioral facial twitching. Neurology was consulted, and she was treated with benzodiazepines and a levetiracetam loading dose followed by maintenance dosing.

Continuous electroencephalography demonstrated severe diffuse encephalopathy but no epileptiform discharges or electrographic seizures. Her myoclonus resolved over the next 48 hours. Serial laboratory studies showed improvement in lactic acidosis and liver enzyme elevations, with creatine kinase peaking and then downtrending. On hospital day two, after a successful ECMO clamp trial and normalization of hemodynamics, she was taken to the operating room for ECMO decannulation with femoral artery and vein repair. Transthoracic echocardiography performed around this time demonstrated normal left ventricular size with preserved systolic function, with an estimated ejection fraction of 50–55% (normal 50–70%) and mildly reduced right ventricular function.

She remained intubated postoperatively but was weaned to pressure support ventilation and extubated on hospital day three. Following extubation, the patient was awake, following commands, and oriented, with no focal neurologic deficits. Her thrombocytopenia and coagulopathy, attributed to ECMO and critical illness, gradually improved. She completed a short course of broad-spectrum antibiotics, participated in physical therapy, and transitioned to an oral diet. She was discharged home on hospital day seven neurologically intact, with close cardiology, neurology, and toxicology follow-up arranged.

## DISCUSSION

This case illustrates the life-threatening cardiac toxicity that may result from ingestion of high-potency kratom products. While mitragynine does not classically exhibit arrhythmogenic properties, case reports and animal studies suggest that it may prolong the QT interval and lead to ventricular dysrhythmias under certain conditions.^2^,^3^,^5^,^6^ Proposed mechanisms include direct sodium and potassium channel inhibition, mitochondrial toxicity, and sympathetic overdrive.^4^,^6^

In our patient, profound hypokalemia likely played a critical role in creating a vulnerable myocardial substrate for arrhythmogenesis. Although the cause of the hypokalemia is unclear, it may have been related to vomiting, catecholamine surge, or renal wasting induced by kratom’s adrenergic effects.^9^ Kratom may also have had a direct impact on pancreatic beta-cell function or insulin sensitivity, which could have further altered electrolyte dynamics and contributed to the development of metabolic acidosis and hypokalemia.^8^

While cardiac arrest in young patients is often attributed to congenital or structural causes, clinicians should also consider toxicologic etiologies, particularly in the absence of underlying heart disease. The increasing availability and use of kratom among young adults underscores the need for greater clinical awareness of its toxic potential. MIT45 Super K Extra Strong is advertised as containing 150–200 mg of mitragynine per bottle, which far exceeds traditional use levels. Additionally, due to the unregulated nature of these supplements, contamination with synthetic substances or adulterants is possible and may enhance toxicity.^5^

Venoarterial extracorporeal membrane oxygenation is not routinely available in all EDs, but its use in select cases of refractory toxicologic cardiac arrest has shown promise.^7^ In this case, VA-ECMO served as a bridge to recovery, allowing for correction of acidosis and electrolyte abnormalities while maintaining end-organ perfusion. Emerging evidence supports the use of extracorporeal life support in poisoned patients with potentially reversible causes of cardiac arrest who do not respond to conventional resuscitation.^7^ Criteria for ECMO consideration often include patients < 65 years of age, witnessed arrest, immediate initiation of CPR with acceptable low-flow duration, and absence of significant comorbidities.^7^ Early activation of the ECMO team is critical when patients meet these criteria.

Given the unpredictable content and potency of commercial kratom products, emergency physicians must maintain a high index of suspicion in cases of unexplained cardiac arrest or seizure, particularly in younger individuals. This case highlights both the dangers of high-potency kratom ingestion and the importance of advanced resuscitative strategies such as VA-ECMO in select patients. Improved public health regulation and product oversight are urgently needed to reduce the risk of life-threatening toxicity from kratom.

## CONCLUSION

Kratom toxicity can manifest with devastating cardiac consequences, particularly when consumed in concentrated liquid formulations. Emergency physicians should consider kratom as a potential etiology in young patients presenting with unexplained seizures, arrhythmias, or cardiac arrest. Profound hypokalemia, severe acidemia, and ventricular dysrhythmias may signal kratom-induced toxicity. Prompt recognition, aggressive supportive care, and early ECMO consideration can be lifesaving. As kratom use continues to rise, broader awareness and regulatory action are necessary to address its growing impact on public health.
